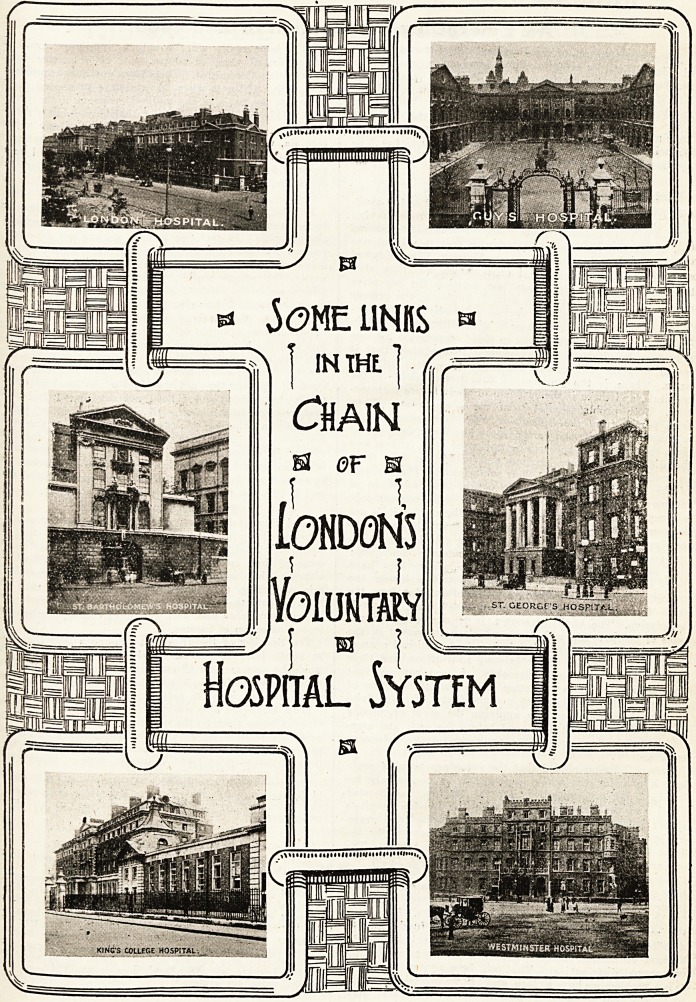# Do the People Understand?

**Published:** 1920-06-26

**Authors:** 


### THE VOLUNTARY SYSTEM.

### Do the People Understand?

Sir Napier Burnett's contribution to the hospital problem is of high potential value. In what must have been a very exhaustive and searching inquiry this gentleman has discovered the wants and the weaknesses of voluntary hospitals, and summarised them with great skill. But if the ascertained facts are not brought home to the public conscience, so that the people are made to understand, very little will have been done towards finding a solution.

### The Subtlety of Socialism.

It is the fashion nowadays to advocate State management, and very telling arguments can be advanced in its favour. The arguments of the uncompromising Socialist possess a subtle attractiveness in that they relieve the individual of all responsibility, and cast the burden on something intangible which has neither body nor soul. The voluntary hospital has arrived at a place where the road bifurcates, and it will immediately have to be decided whether the taxpayer or the philanthropist is to carry on the work of healing. The word "philanthropist" is not here used in the conventional sense, which confines its application to the wealthy, but in its wider signification, which includes everybody, rich or poor, who responds to the command, "Thou shalt love thy neighbour as thyself."

This is not a homily. I am not given to preaching, nor to autobiography. But one's personal experience may be valuable and void of egotism. When the war broke out I was a convinced believer in the State machine. So were vast crowds, who like myself afterwards became converted to another creed. We had a State Department which declared war. We had the Admiralty and the War Department for making war, and a Revenue Department for imposing the cost on the nation. We had, moreover, a Government with plenipotentiary powers of conscription.

There was absolutely nothing missing. And yet, looking backward, I am as convinced as I am of my own existence that but for voluntary civilian effort the war would have been lost. Tears and treasure were poured forth without stint. Prince and peasant, youths and maidens, old men creeping with their shadows, children with their sunny eyes—all of them followed the Red Cross beacon which lighted the way to victory.

We are all Socialists, more or less. If the truth were known, probably our most Conservative thinkers would be found to be the most out-and-out Socialists. Nomenclature counts for more than people wot of. There is a vast chasm between the Socialist who realises responsibility and the Socialist who seeks to shift it.

My point is that the people need education. They do not understand. They did not understand what the Red Cross meant when the world decided to engage in war. The truth dawned very gradually. And it did not dawn in any automatic or mysterious manner corresponding with sundawn. The truth had to be preached. Men, women, and children had to be taught that on their efforts and on their attitude depended victory.

The voluntary system, if continued, will have to be thoroughly reorganised. It is perfectly clear from Sir Napier Burnett's able report that it has come a cropper. Hospital boards have not been able to adjust their requirements to war-time changes. Reform, it is obvious, must begin with the nurses. There is no occupation that more powerfully appeals to a woman than nursing, and it should cause hospital governors very seriously to think when it is placed on record after searching and impartial investigation that "a spirit of revolt against the long hours of drudgery in ward work and the low rates of pay that have hitherto obtained" is apparent. It is high time that this fact should be chronicled. The nurse was made a slave and her claims relegated to silence. The result is that the high priestess of the temple is disappearing. There is no fuss. She is simply deserting the profession and advising others to do likewise. Sir Napier does not mince his words. Revolt is in the air, and is the outcome of neglect. For years she was dazzled with the prospect of salvation through registration. But good salaries and better conditions of service do not necessarily follow the keeping of a name register at Westminster.

The subject is large and complex. **I believe the true solution lies in making the people understand.**

IERNE.

### One Year's Roll-Call of the Sick.

**Patients Treated at Metropolitan Voluntary Hospitals and Dispensaries and M.A.B. Infectious Hospitals.**

| | |
|---|---|
| Sufferers needing Surgical Aid | 605,803 |
| Sufferers needing Medical Care | 556,822 |
| Sufferers from Eye Troubles | 155,462 |
| Diseases of Women | 99,058 |
| Diseases of the Ear, Nose, and Throat | 73,235 |
| Sufferers from Skin Diseases | 63,520 |
| Consumptives | 44,542 |
| Fever Patients | 23,999 |
| Paralysis and Epilepsy | 23,153 |
| **Total** | **1,645,594** |

The above figures include only the in-patient cases treated to a termination in the wards of the hospitals and the number of new out-patient cases treated in the out-patient departments and dispensaries; 172,209 of these patients were children. They claim our sympathy and help. To the vigorous, to those in health who are able to provide for their dependants, to those who know what ill-health means, who have suffered from disease of one kind or another, and who, either in the hospital or under the skill and care of the doctors and nurses trained in the hospitals, have been restored to health and usefulness, we confidently appeal on behalf of the London hospitals.

LONDON HOSPITAL.

GUY'S HOSPITAL.

### SOME LINKS IN THE CHAIN OF LONDON'S VOLUNTARY HOSPITAL SYSTEM

ST. BARTHOLOMEW'S HOSPITAL.

ST. GEORGE'S HOSPITAL.

## Figures and Tables

**Figure f1:**